# Concordance and Relative Performance of the 2023 ACR/EULAR and Revised Sapporo Criteria in Antiphospholipid Syndrome and Analysis of Risk Factors for Recurrent Thrombosis: A Single-Center Cohort Study

**DOI:** 10.3390/medicina62071226

**Published:** 2026-06-24

**Authors:** Mehmet Akif Baltaci, Emine Gozde Aydemir Guloksuz, Ruveyda Sak Inal, Ayse Tugcenur Temiz Gencoglu, Gulnur Celik Yilmaz, Enver Caner Ceran, Samet Dal, Tugce Elmali Yazli, Recep Yilmaz, Asli Ciftci, Aslihan Avanoglu Guler, Hakan Apaydin, Cem Ozisler, Alper Sari, Sevinc Can Sandikci, Melih Pamukcu, Ayse Bahar Kelesoglu Dincer

**Affiliations:** 1 Department of Rheumatology, Ankara Etlik City Hospital, 06170 Ankara, Turkey; 2 Department of Internal Medicine, Ankara Etlik City Hospital, 06170 Ankara, Turkey; 3 Sincan Training and Research Hospital, Ministry of Health, 06932 Ankara, Turkey; 4 Department of Biostatistics, Ankara University, 06230 Ankara, Turkey; 5 Division of Rheumatology, Department of Internal Medicine, Ufuk University Hospital, Ufuk University Faculty of Medicine, 06520 Ankara, Turkey

**Keywords:** 2023 ACR/EULAR classification criteria, antiphospholipid syndrome, revised Sapporo criteria, sensitivity and specificity, recurrent thrombosis

## Abstract

*Background and Objectives:* To evaluate the concordance and relative performance of the 2023 American College of Rheumatology (ACR)/European Alliance of Associations for Rheumatology (EULAR) classification criteria compared with the revised Sapporo (2006) criteria and to identify potential risk factors for recurrent thrombosis among patients classified as antiphospholipid syndrome (APS) according to the 2023 criteria. *Materials and Methods:* This retrospective cohort study included 82 patients with documented antiphospholipid antibody (aPL) positivity between October 2022 and August 2024. The revised Sapporo criteria were used as the comparator classification system to evaluate the concordance and relative performance of the 2023 ACR/EULAR criteria. Secondary analyses were restricted to patients fulfilling the 2023 criteria (n = 55), in whom clinical characteristics and potential risk factors for recurrent thrombosis were evaluated. *Results:* Of the 82 aPL-positive patients, 9 fulfilled only the revised Sapporo criteria, 1 fulfilled only the 2023 criteria, 54 met both criteria sets, and 18 met neither. Using the revised Sapporo criteria as the comparator, the 2023 criteria demonstrated a relative sensitivity of 85.7% and relative specificity of 95.0%. Positive and negative agreement rates were 98.2% and 66.7%, respectively. Among patients classified as APS according to the 2023 criteria, disease duration was significantly longer in patients with recurrent thrombosis in univariate analysis (*p* = 0.028); however, this association did not remain significant after multivariable adjustment. *Conclusions:* The 2023 ACR/EULAR criteria demonstrated higher relative specificity but lower relative sensitivity compared with the revised Sapporo criteria. No independent predictor of recurrent thrombosis was identified after multivariable adjustment, although the limited number of outcome events may have reduced statistical power. However, most recurrent thrombotic events occurred in patients with subtherapeutic INR levels, suggesting the importance of careful anticoagulation management in patients with APS.

## 1. Introduction

Antiphospholipid syndrome (APS) is a systemic autoimmune disorder characterized by macro/micro-vascular thrombosis, pregnancy morbidity, and non-thrombotic manifestations in patients with persistently positive antiphospholipid antibodies (aPL) on ≥2 occasions 12 weeks apart [1]. It is one of the most common causes of acquired thrombophilia affecting both the arterial and venous circulations, and primarily presents in young adults [2].

The first classification criteria for APS were proposed in Sapporo in 1999 and subsequently revised in 2006 during a consensus meeting in Sydney, Australia. According to the revised Sapporo criteria, patients have been classified as having APS when they have had at least one clinical feature of thrombosis or pregnancy morbidity together with persistent antiphospholipid antibody positivity on two or more occasions at least 12 weeks apart [3,4]. However, the revised Sapporo criteria had several limitations, as non-thrombotic manifestations were not included in the criteria (i.e., non-criteria manifestations), and the established risk factors for venous thromboembolism, as well as the cardiovascular risk factors for arterial thrombosis, were not taken into account. These limitations have led to the development of updated classification criteria. In 2023, with the collaboration of the American College of Rheumatology (ACR) and the European Alliance of Associations for Rheumatology (EULAR), new classification criteria incorporating a weighted scoring system based on an expanded set of clinical features, including macrovascular, microvascular, obstetric, cardiac valvular, and hematologic domains, as well as refined aPL profiles, were introduced. Moreover, in the 2023 ACR/EULAR classification criteria, vascular thrombosis risk factors were incorporated into the classification framework and specified separately for arterial and venous thrombosis [1]. As part of the laboratory domain modifications, a distinction was made between IgG and IgM isotypes of anticardiolipin (aCL) and anti-β2-glycoprotein I (aβ2GPI) antibodies, with isolated IgM positivity assigned a lower weight. Furthermore, thresholds for moderate (40–79 units) and high (>80 units) positivity were defined for both aCL and aβ2GPI antibodies [1].

Despite advances in classification and treatment, thrombotic events remain the most frequent clinical manifestations and the leading cause of mortality in APS, with a 10-year survival rate estimated to be 90.7% [5]. The rate of recurrent thrombosis in APS patients was shown to be approximately 17% in the first five years, and 14% in the subsequent five years [6]. In a recently published study from Brazil, including 155 primary APS patients, an incidence rate of 3.4 per 100 patient-years was reported for recurrent thrombosis [7]. Previous studies have identified several factors associated with increased risk of recurrence, including arterial thrombosis, triple aPL positivity, persistent antibody positivity, and coexisting autoimmune diseases—especially systemic lupus erythematosus (SLE) [8]. Therefore, early identification of high-risk patients is essential. In order to predict the risk of thrombosis, the Global APS Score (GAPSS) was initially developed in patients with SLE and later validated in primary APS [9]. Since the GAPSS includes anti-phosphatidylserine/prothrombin (anti-PS/PT) antibodies, which are not routinely measured in clinical practice, a simplified and adjusted version of the score (aGAPSS), excluding anti-PS/PT, was subsequently developed and validated [10]. The adjusted Global Antiphospholipid Syndrome Score has emerged as a useful tool for risk stratification, combining aPL profiles with conventional cardiovascular risk factors such as arterial hypertension and dyslipidemia [11].

The primary goal of this study was to assess the concordance and relative performance of the 2023 ACR/EULAR classification criteria in comparison with the revised 2006 Sapporo criteria. The secondary objectives were to characterize the clinical and laboratory features of patients classified as APS according to the 2023 ACR/EULAR classification criteria and to identify potential risk factors for recurrent thrombosis.

## 2. Materials and Methods

### 2.1. Patient Selection

Patients with documented aPL positivity were retrospectively enrolled at the Rheumatology Department of Ankara Etlik City Hospital between October 2022 and August 2024. All patients were evaluated according to the revised Sapporo (Sydney) criteria, which served as the comparator classification system for APS classification. The 2023 ACR/EULAR APS classification criteria were then applied to the same cohort, and their relative sensitivity and relative specificity were calculated against the revised Sapporo criteria. For secondary analyses, only patients classified as APS according to the 2023 ACR/EULAR criteria were included, while the remaining patients were excluded from these analyses.

Arterial hypertension was defined as blood pressure ≥140/90 mmHg on at least two separate occasions or current use of oral antihypertensive medication [12]. Hyperlipidemia was defined as low-density lipoprotein cholesterol (LDL-C) ≥160 mg/dL and/or triglycerides ≥175 mg/dL on at least two measurements, or current use of statin therapy [13].

Duration of disease was defined as the time from the diagnosis of APS to the time of the last clinical visit. Recurrent thrombosis was defined as any new arterial, venous, or microvascular thrombotic event occurring after the index thrombotic event. All recurrent events were documented through appropriate imaging studies (Doppler ultrasound, computed tomography, or magnetic resonance imaging) and by reviewing hospital records, including comparison with previous imaging when available.

Demographic, clinical (macrovascular, microvascular, obstetric, cardiac valvular, and hematologic involvements as defined in the 2023 ACR/EULAR classification criteria), and laboratory data were retrospectively collected through the hospital’s electronic medical record system.

### 2.2. Laboratory and aGAPSS

The aPL profile included lupus anticoagulant (LA), aCL IgG/IgM, and aβ2GPI IgG/IgM antibodies. aCL IgG/IgM and aβ2GPI IgG/IgM antibodies were measured using enzyme-linked immunosorbent assay (ELISA), and positivity was defined as titers >40 GPL or MPL units or above the 99th percentile. In accordance with the 2023 ACR/EULAR APS classification criteria, titers were further categorized as moderate (40–79 GPL/MPL units) and high (≥80 GPL/MPL units or >99th percentile). Lupus anticoagulant (LA) was assessed in accordance with the updated ISTH guidelines, using a dRVVT-based screen/confirm assay (Lupus S and Lupus C; Roche Diagnostics GmbH, Mannheim, Germany) complemented by an LA-sensitive aPTT. All analyses were performed on the cobas t 511/t 711 coagulation analyzer (Roche Diagnostics GmbH, Mannheim, Germany), and LA positivity was defined as a normalized screen/confirm ratio >1.2, based on the manufacturer’s 99th-percentile cut-off. Antiphospholipid antibody positivity was reassessed in all patients at an interval of at least 12 weeks in accordance with the revised Sapporo criteria. Both the revised Sapporo criteria and the 2023 ACR/EULAR APS classification criteria were applied to all participants.

aGAPSS was calculated as previously described, by assigning weighted points to each risk factor: 3 points for hyperlipidemia, 1 point for arterial hypertension, 5 points for aCL IgG/IgM positivity, 4 points for aβ2GPI IgG/IgM positivity, and 4 points for LA positivity. The maximum possible score for each patient was 17 [11].

### 2.3. Statistical Analysis

Descriptive statistics were presented as mean ± standard deviation (SD) or median (minimum–maximum) for continuous variables, and as frequencies and percentages for categorical variables. The normality of data distribution was assessed using the Kolmogorov–Smirnov test. For categorical variables, comparisons were performed using the chi-square test or Fisher’s exact test, as appropriate. Differences between two groups were analyzed using the Mann–Whitney U test for non-normally distributed variables, while comparisons among three groups were conducted using the Kruskal–Wallis test. To describe the performance characteristics of the parameters relative to the revised Sapporo criteria, relative sensitivity, relative specificity, positive predictive value (PPV), and negative predictive value (NPV) were calculated. Cohen’s kappa coefficient was also calculated to assess the degree of agreement between the two classification systems. Multivariable logistic regression analysis was performed to evaluate factors independently associated with recurrent thrombosis. To assess the robustness of the multivariable model and address potential small-sample bias related to the limited number of recurrent thrombotic events, a sensitivity analysis using Firth’s bias-reduced logistic regression was additionally performed using the same set of covariates. Variables included in the multivariable model were selected based on clinical relevance, prior evidence from the literature, and the results of univariate analyses. Disease duration was included because it was significantly associated with recurrent thrombosis in univariate analysis, whereas age and aGAPSS were included based on their established clinical relevance and previous literature suggesting an association with thrombotic risk in APS. Standard statistical analyses were performed using SPSS for Windows (version 30.0; IBM Corp., Armonk, NY, USA), and a sensitivity analysis using Firth’s bias-reduced logistic regression was performed in R (version 4.6.0; R Foundation for Statistical Computing, Vienna, Austria) using the logistf package. *p*-values <0.05 were considered statistically significant.

## 3. Results

A total of 82 patients with aPL positivity were evaluated. Of the 82 patients, 9 were classified with only the revised Sapporo criteria, one with only the 2023 ACR/EULAR APS criteria, 54 with both criteria sets, and 18 with neither. When the revised 2006 Sapporo criteria were used as the comparator classification system, the 2023 ACR/EULAR criteria demonstrated a relative sensitivity of 85.7% (95% CI, 75.0–92.3), relative specificity of 95.0% (95% CI, 75.4–99.1), positive agreement of 98.2% (95% CI, 90.4–99.7), and negative agreement of 66.7% (95% CI, 47.8–81.4) (Table 1). Cohen’s kappa coefficient was 0.70 (95% CI, 0.53–0.88), indicating substantial agreement between the two classification systems.

As part of the secondary objective of the current study, 27 patients from the total cohort were excluded, as they were not classified as APS according to the 2023 ACR/EULAR criteria. Of these, nine had isolated aPL positivity and did not meet the entry criteria, while the remaining eighteen fulfilled the entry criteria but did not reach the required clinical and laboratory scores for APS classification. The clinical events and antibody status of these 18 patients are presented in Table 2. Thus, 55 patients who met the new classification criteria for APS were included in the subsequent analyses. The mean age was 39.64 ± 12.22 years, and the majority were female (38, 69.1%). The median disease duration was 12 months. Thirty-one patients (56.4%) were classified as having secondary APS, and among them, 24 (77.4%) had SLE. Among the 55 patients classified as APS, 43 (78.2%) were receiving anticoagulation therapy, the majority of whom were treated with vitamin K antagonists (34/43, 79.1%). Demographic and treatment features were summarized in Table 3.

The most common clinical manifestation was venous thromboembolism (VTE), observed in 33 patients (60.0%), followed by arterial thrombosis, which occurred in 24 patients (43.6%). Among the cohort, obstetric complications occurred in 20 patients (36.3%), and thrombocytopenia was identified in 19 patients (34.5%). Four (7.4%) patients had one-time LA positivity, and 43 (79.6%) had persistent LA positivity. A total of 26 patients (47.2%) had moderate and/or high positive IgG antiphospholipid antibody levels (aCL and/or aβ2GPI). Low C3 levels were detected in 8 patients (14.5%), while both C3 and C4 were decreased in 4 patients (7.2%). Table 4 summarizes the laboratory and clinical characteristics of the patients in detail.

When the comparison was made between patients with (n = 17) and without (n = 38) recurrent thrombosis, disease duration was significantly longer in patients with recurrent thrombosis (*p* = 0.028) (Table 5). However, this association did not remain significant after adjustment for age and aGAPSS in multivariable logistic regression analysis (Table 6). A sensitivity analysis using Firth’s bias-reduced logistic regression, performed with the same covariates, yielded results consistent with those of the standard multivariable model, and no independent predictors of recurrent thrombosis were identified. Among 17 patients with a history of recurrent thrombosis, all of whom were receiving warfarin therapy, the INR value obtained at the time of the thrombotic event was available. Ten patients had an International Normalized Ratio (INR) below 2.0 at the time of recurrence. Notably, two patients experienced recurrent thrombotic events despite having INR values within the therapeutic range (2.0–3.0). In both cases, the prior thrombotic event had been venous in nature, and an INR target of 2.0–3.0 therefore reflected the appropriate therapeutic range for these patients.

**Figure 1:**
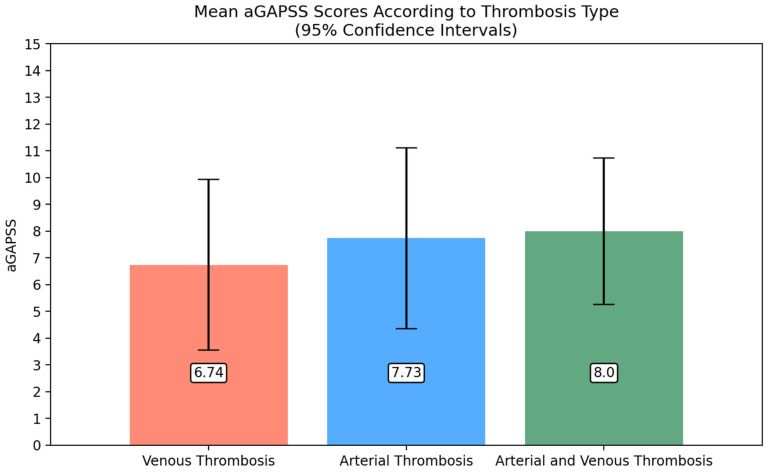
Mean aGAPSS scores according to thrombosis type. Bars represent mean values and whiskers indicate 95% confidence intervals. Kruskal–Wallis test, *p* = 0.325.

Comparison of aGAPSS among patients with arterial thrombosis only, venous thrombosis only, and both arterial and venous thrombosis revealed no statistically significant differences (*p* = 0.325) (Figure 1). In case of thrombotic recurrence, whether the recurrent event was arterial or venous, no significant difference in aGAPSS scores was observed compared to patients with a single thrombotic event (6.53 ± 2.62 vs. 8.00 ± 3.57, *p* = 0.095) (Table 5).

## 4. Discussion

In this study, we showed that the relative sensitivity and relative specificity of the 2023 ACR/EULAR classification criteria for APS are 85.7% (95% CI, 75.0–92.3), and 95.0% (95% CI, 75.4–99.1), respectively when compared to the revised Sapporo criteria. However, the confidence interval around the relative specificity estimate was notably wide (95% CI, 75.4–99.1), reflecting limited precision attributable to the modest number of patients who did not meet either classification criteria, and should therefore be interpreted with caution. Accordingly, in a cohort of 82 patients with aPL-positivity, 55 patients were classified as APS with the new classification criteria. Venous thromboembolism was the most common vascular manifestation, followed by arterial thrombosis. Although longer disease duration was associated with recurrent vascular events in univariate analysis, this association did not remain significant after multivariable adjustment within this cohort, which should be interpreted in the context of the limited number of outcome events.

The main goal of developing the 2023 ACR/EULAR APS classification criteria was to improve the specificity and achieve a more homogeneous patient cohort for research purposes compared to the revised Sapporo criteria. Since the introduction of the 2023 ACR/EULAR APS classification criteria, several studies have been published that evaluated the relative performance and validation of the new criteria compared to the revised Sapporo criteria. Using the revised Sapporo criteria as the comparator classification system, Vasi et al. found the 2023 ACR/EULAR APS criteria to have a relative sensitivity of 82.5% and a relative specificity of 98%; Usta et al. reported 77% and 97.6%, respectively [14,15]. Zhao et al. evaluated the 2023 APS classification criteria in their APS cohort, reporting a relative sensitivity of 81.8% and a relative specificity of 97.6%, while Estefanía et al. similarly demonstrated increased relative specificity (97.7%) in their Spanish cohort [16,17]. Using the revised Sapporo criteria as the comparator classification system, in this current study, we found that the 2023 ACR/EULAR APS classification criteria had a relative sensitivity of 85.7% and a relative specificity of 95%. Consistent with the aforementioned studies, we also showed that the 2023 ACR/EULAR criteria had higher relative specificity but lower relative sensitivity when benchmarked to the revised Sapporo criteria.

In our cohort of 82 patients, 18 met the 2023 ACR/EULAR entry criteria but did not achieve the minimum clinical and laboratory scores required for APS classification; however, 9 of these 18 patients were still classified as APS according to the revised Sapporo criteria (patients no. 1–9 on Table 2). Among those nine patients, five failed to meet the clinical component, two failed to meet the laboratory component, and two failed to meet both. All seven patients who did not meet the clinical criteria presented with obstetric complications. The strict scoring of obstetric items in the 2023 ACR/EULAR criteria reduced concordance with the Sapporo criteria, thereby lowering the 2023 ACR/EULAR criteria’s overall sensitivity. This finding was anticipated and is consistent with previous reports in the literature [14,15,16,18]. In the study by Vasi et al., all six patients who were classified according to the revised Sapporo criteria but had insufficient clinical scores according to the 2023 ACR/EULAR classification criteria presented with obstetric complications [14]. Similarly, Zhao et al. reported sixteen patients who met the pregnancy morbidity component of the revised Sapporo criteria but could not be classified according to the 2023 ACR/EULAR classification criteria, while Estefanía et al. found that 55 (85.9%) of 64 obstetric APS patients fulfilling the revised Sapporo criteria did not meet the new criteria [16,17]. Kwon et al. reported a concordance rate of 84.9% between the revised Sapporo criteria and the 2023 ACR/EULAR classification criteria, which varied according to the index event, with the lowest agreement observed among patients with obstetric events (45.5%) [18]. While at least three unexplained spontaneous abortions before 10 gestational weeks, early fetal loss (10 weeks–15 weeks 6 days), or fetal death in the absence of severe pre-eclampsia (PEC) or severe placental insufficiency (PI) (16 weeks 0 days–33 weeks 6 days) were sufficient for the 2006 revised Sapporo criteria, these pregnancy complications alone are not sufficient to fulfill the 2023 ACR/EULAR APS classification criteria [1,4]. These findings highlight the need for careful clinical evaluation of patients with obstetric manifestations who do not fulfill the 2023 criteria, as such patients may still be at risk for adverse pregnancy outcomes and may benefit from individualized management.

The updates to the 2023 laboratory criteria appear to have reduced sensitivity while enhancing specificity. According to the 2006 revised Sapporo criteria, the presence of moderate or high titers of IgG and/or IgM aCL or aβ2GPI antibodies detected by ELISA was sufficient for classification. In contrast, the 2023 ACR/EULAR criteria place greater emphasis on persistence and antibody type, requiring sustained LA positivity and/or moderate (40–79 units) to high (≥80 units) titers of IgG aCL or aβ2GPI antibodies, while single LA or isolated IgM positivity has been considered insufficient [1,4]. In our study, aCL and aβ2GPI IgM positivity was detected in four patients who did not meet the laboratory criteria. Isolated IgM positivity can occur in conditions such as infections, malignancies, and drug use [19,20,21]. However, isolated aCL and/or aβ2GPI IgM can be detected, particularly in obstetric APS [22]. In an algorithm predicting pregnancy morbidity in women with persistent aPL positivity, Pregnolato et al. demonstrated that low-titer aPL significantly increased the risk of pregnancy morbidity, with approximately 40% of these antibodies being of the IgM isotype [23]. It should also be noted that non-criteria aPL may be present in some patients who do not fulfill current classification criteria despite having clinical features suggestive of APS. Although these markers were not assessed in the present cohort, they may partly explain discordant classification results in selected patients and warrant further investigation in future studies [24].

Among 55 patients classified according to the 2023 ACR/EULAR criteria, primary APS was present in 44.6%, whereas SLE was identified in 77.4% of those with secondary APS. Our relatively small number of primary APS cases, compared with the literature [5,25], could be explained by both the limited study population and the referral characteristics of our center, as patients with thrombosis or pregnancy morbidity in the absence of other rheumatological findings are often managed outside rheumatology clinics.

Thrombocytopenia, included as a clinical domain in the new criteria, is among the most frequent hematologic manifestations of APS, reported in 22–42% of patients [26]. The 34.5% rate observed in our cohort aligns well with previous data, supporting the representativeness of our study population. Thrombocytopenia has been linked to an increased risk of recurrent thrombosis, obstetric complications, and severe non-thrombotic manifestations [27]. Its inclusion in the new criteria may help recognize a broader spectrum of APS presentations. However, it is essential to distinguish whether thrombocytopenia is a direct manifestation of APS or secondary to an underlying autoimmune disease. Notably, patients presenting with thrombocytopenia and positive aPL antibodies alone cannot be classified as having APS [1]. In our study, among the 16 patients who met the 2023 ACR/EULAR entry criteria but did not achieve the clinical score required for classification, thrombocytopenia was the only clinical domain in half of the cases. All of these patients had concomitant autoimmune diseases, predominantly SLE. In addition, the new criteria define thrombocytopenia within a platelet range of 20 × 10^9^/L to 130 × 10^9^/L, thereby excluding values below 20 × 10^9^/L [1]. This implies that, although uncommon, APS patients with more severe thrombocytopenia (<20 × 10^9^/L) may not meet the classification criteria.

Seventeen of the 55 patients included in the study developed recurrent thrombosis during the follow-up. In univariate analysis, disease duration was significantly longer in patients with recurrent thrombosis. However, this association did not remain significant after adjustment for age and aGAPSS in multivariable analysis. In a study of 379 patients based on data from the APS-ACTION clinical database, hypertension, hyperlipidemia, diabetes, age, smoking, and the presence of LA, aCL IgG/IgM, aβ2GPI IgG/IgM, or triple antibody positivity were reported not to be significant risk factors for recurrent thrombosis [11]. Although that study found no independent association between isolated aPL positivity or traditional cardiovascular risk factors and the risk of recurrent thrombosis, patients with recurrent events had significantly higher aGAPSS scores (*p* < 0.05) [11]. In our cohort, however, we did not observe a significant relationship between aGAPSS and recurrent thrombosis (*p* = 0.095). This difference may be explained by the smaller sample size of our cohort, the lower prevalence of hyperlipidemia (5.9% vs. ~30%), the younger mean age of our patients (41.8 ± 11.9 vs. 50 ± 12 years), and the lower proportion of triple antibody–positive cases (0% vs. 17%), a finding that is contrary to the existing literature and most likely reflects the small sample size rather than a true biological difference; therefore, it should not be overinterpreted. The younger age of our cohort likely contributed to fewer comorbid cardiovascular risk factors such as diabetes, hypertension, and hyperlipidemia, which may have influenced the overall aGAPSS distribution, as age itself is not directly included in the score. Additionally, the limited number of recurrent thrombotic events in our cohort may have contributed to this finding. Nevertheless, in a recent study by Zen et al., consistent with our findings, the 2023 ACR/EULAR clinical domains were not associated with an increased risk of recurrent thrombosis [28]. Inadequate anticoagulation in patients with APS may contribute to the risk of recurrent thrombosis. In our cohort, only two patients developed thrombosis while their INR values were within the therapeutic range, whereas the remaining patients were below target levels at the time of the thrombotic event.

There are several limitations of this study that should be acknowledged. The main limitations include its retrospective design, relatively small sample size (n = 55), and single-center setting, which may restrict the generalizability of the findings. Furthermore, the high proportion of secondary and SLE-associated APS in our cohort likely reflects referral bias inherent to a tertiary rheumatology center, where patients with established connective tissue diseases are preferentially referred, and may limit the applicability of our findings to the broader APS population. In addition, the absence of a non-APS control group and the inclusion of exclusively aPL-positive patients may have limited our ability to comprehensively assess the relative specificity of the 2023 ACR/EULAR criteria compared with the revised Sapporo criteria. Consequently, the reported performance measures may not be generalizable to unselected patients undergoing APS evaluation in broader clinical settings. Because both the revised Sapporo criteria and the 2023 ACR/EULAR criteria are classification criteria rather than diagnostic standards, the reported performance measures should be interpreted as relative comparisons between classification systems rather than true estimates of diagnostic accuracy. The use of one classification system as a comparator for another introduces an inherent circularity, and the resulting performance measures should therefore be interpreted with caution. Differences in follow-up duration between patients may have affected both the evaluation of recurrent thrombosis and the observed associations. Furthermore, detailed follow-up data, including median follow-up time and total patient-years of observation, were not available for the current analysis, limiting the interpretation of recurrence rates and precluding the calculation of incidence rates. Future studies incorporating standardized follow-up data and time-to-event analyses may provide a more robust assessment of risk factors for recurrent thrombosis. Although multivariable logistic regression analysis was performed, the limited number of recurrent thrombotic events may have reduced statistical power and increased the risk of overfitting. Therefore, the absence of statistically significant associations should not be interpreted as evidence that clinically relevant risk factors do not exist. All findings regarding recurrent thrombosis should be interpreted with appropriate caution. Nevertheless, our study provides real-world data contributing to the ongoing evaluation of the 2023 ACR/EULAR classification criteria and offers additional insight into recurrent thrombosis in APS patients.

## 5. Conclusions

In conclusion, the 2023 ACR/EULAR classification criteria demonstrated higher relative specificity but lower relative sensitivity compared with the 2006 revised Sapporo criteria. This reduced sensitivity underscores the need for careful evaluation of patients with pregnancy morbidity who do not fully meet the clinical criteria, as well as those with single-time LA positivity or isolated IgM aPL positivity. In addition, although disease duration was associated with recurrent thrombosis in univariate analysis, this association did not remain significant after multivariable adjustment, and further studies are needed to better define predictors of recurrent thrombosis in APS. Nevertheless, a considerable proportion of patients with recurrent thrombosis had subtherapeutic INR levels at the time of the thrombotic event. Overall, these findings suggest the importance of thorough patient evaluation, individualized risk stratification, and strict anticoagulation management to improve outcomes in patients with APS.

## Figures and Tables

**Table 1:** Relative Performance and Agreement Measures of the 2023 ACR/EULAR Classification Criteria Compared with the Revised Sapporo Criteria.

Parameter	Value	95% Confidence Interval
Disease prevalence	0.77	
Relative sensitivity	0.85	0.75–0.92
Relative specificity	0.95	0.75–0.99
Positive predictive value (PPV)	0.98	0.90–0.99
Negative predictive value (NPV)	0.67	0.48–0.81
Cohen’s kappa coefficient	0.70	0.53–0.88

Note: Values are presented as proportions. Abbreviations: PPV, positive predictive value; NPV, negative predictive value.

**Table 2:** Clinical Characteristics and Antibody Profiles of Patients Meeting the Entry Criteria but not Classified According to the 2023 ACR/EULAR Classification Criteria.

	Clinical Features	Laboratory Features	2023 ACR/EULAR Scores	2023 ACR/EULAR APS Criteria Status
Patient 1	≥3 consecutive pre-fetal (<10w) death	Moderate positive IgG (aCL and aβ2GPI) and persistent positive LA	Clinical: 1 Laboratory: 9	Clinical domain score is inadequate
Patient 2	Fetal death (16w–33w 6d) in the absence of PEC or PI	Moderate positive IgG (aCL and aβ2GPI) and one-time positive LA	Clinical: 1 Laboratory: 5	Clinical domain score is inadequate
Patient 3	Early fetal death (10w–15w 6d)	Persistent positive LA	Clinical: 1 Laboratory: 5	Clinical domain score is inadequate
Patient 4	Early fetal death (10w–15w 6d) and fetal death (16w–33w 6d) in the absence of PEC or PI	Persistent positive LA	Clinical: 2 Laboratory: 5	Clinical domain score is inadequate
Patient 5	VTE without a high-risk VTE profile	Moderate positive IgM (aCL and aβ2GPI)	Clinical: 3 Laboratory: 1	Laboratory domain score is inadequate
Patient 6	Arterial thrombosis without a high-risk CVD profile	Moderate positive IgM (aCL and aβ2GPI)	Clinical: 4 Laboratory: 1	Laboratory domain score is inadequate
Patient 7	Early fetal death (10w–15w 6d)	Moderate positive IgM (aCL and aβ2GPI)	Clinical: 1 Laboratory: 1	Clinical and laboratory domain scores are inadequate
Patient 8	≥3 consecutive pre-fetal (<10w) death	Moderate positive IgG (aCL and aβ2GPI)	Clinical: 3 Laboratory: 4	Clinical domain score is inadequate
Patient 9	Early fetal death (10w–15w 6d)	Moderate positive IgM (aCL and aβ2GPI)	Clinical: 1 Laboratory: 1	Clinical and laboratory domain scores are inadequate
Patient 10	Thrombocytopenia	One-time positive LA	Clinical: 2 Laboratory: 1	Clinical and laboratory domain scores are inadequate
Patient 11	Thrombocytopenia	Moderate positive IgG (aCL and aβ2GPI)	Clinical: 2 Laboratory: 4	Clinical domain score is inadequate
Patient 12	Thrombocytopenia	Moderate positive IgG (aCL and aβ2GPI)	Clinical: 2 Laboratory: 4	Clinical domain score is inadequate
Patient 13	Thrombocytopenia	Moderate positive IgG (aCL and aβ2GPI)	Clinical: 2 Laboratory: 4	Clinical domain score is inadequate
Patient 14	Livedo Racemosa	Moderate positive IgG (aCL and aβ2GPI)	Clinical: 2 Laboratory: 4	Clinical domain score is inadequate
Patient 15	Thrombocytopenia	Moderate positive IgG (aCL and aβ2GPI)	Clinical: 2 Laboratory: 4	Clinical domain score is inadequate
Patient 16	Thrombocytopenia	Moderate positive IgG (aCL and aβ2GPI)	Clinical: 2 Laboratory: 4	Clinical domain score is inadequate
Patient 17	Thrombocytopenia	Moderate positive IgM (aCL and aβ2GPI)	Clinical: 2 Laboratory: 1	Clinical and laboratory domain scores are inadequate
Patient 18	Thrombocytopenia	Moderate positive IgM (aCL and aβ2GPI)	Clinical: 2 Laboratory: 1	Clinical and laboratory domain scores are inadequate

Abbreviations: aCL, anticardiolipin antibody; APS, antiphospholipid syndrome; β2GPI, anti-β2-glycoprotein I; LA, lupus anticoagulant; PEC, Preeclampsia; PI, placental insufficiency.

**Table 3:** Demographic and Treatment Features of APS Patients Classified According to the 2023 ACR/EULAR Criteria.

	All Patients (n = 55)
Age (years)	39.64 ± 12.22
Female gender, n (%)	38 (69.1)
Age at diagnosis (years)	35.5 ± 11.9
Disease duration (months)	12 (12–84)
APS group, n (%)	
Secondary APS	31 (56.4)
Systemic lupus erythematosus	24 (77.4)
Undifferentiated connective tissue disease	5 (16.1)
Sjögren’s syndrome	2 (6.5)
Smoking, n (%)	15 (27.2)
Treatment, n (%)	
Hydroxychloroquine	39 (70.9)
Acetylsalicylic acid	18 (32.7)
Warfarin	34 (61.8)
Low-molecular-weight heparin	9 (16.3)

Note: Data are presented as mean ± SD, median (min-max), or number (%), as appropriate. Abbreviations: APS, antiphospholipid syndrome.

**Table 4:** The Clinical and Laboratory Features of APS Patients Classified According to The 2023 ACR/EULAR Criteria.

	All Patients (n = 55)
Clinical domain, n (%)	
Venous thromboembolism	33 (60)
With a high VTE risk profile	4 (7.2)
Without a high VTE risk profile	29 (52.7)
Arterial thrombosis	24 (43.6)
With a high CVD profile	3 (5.4)
Without a high CVD profile	21 (38.1)
Microvascular involvement	
Livedo Racemosa (suspected or established)	2 (3.6)
Livedoid vasculopathy (suspected or established)	7 (12.7)
aPL Nephropathy	2 (3.6)
Pulmonary hemorrhage	1 (1.8)
Myocardial Disease	0
Obstetric	
≥3 consecutive losses (<10 weeks) and/or fetal death(<16 weeks)	6 (10.9)
≥1 fetal death (16–34 weeks) without PEC/PI with severe features	10 (18.1)
Severe PEC or severe PI (<34w)	3 (5.4)
Severe PEC and severe PI (<34w)	1 (1.8)
Cardiac valve	
Thickening	1 (1.8)
Vegetation	4 (7.3)
Hematology	
Thrombocytopenia (20–130 g/L)	19 (34.5)
Recurrent thrombosis, n (%)	17 (30.9)
Laboratory results, n (%)	
Positive LA (One time)	4 (7.4)
Positive LA (Persistent)	43 (79.6)
Moderate or high positive (IgM) (aCL and/or aβ2GPI)	6 (11.1)
Moderate positive (IgG) (aCL and/or aβ2GPI)	15 (27.8)
High positive (IgG) (aCL or aβ2GPI)	4 (7.4)
High positive (IgG) (aCL and aβ2GPI)	7 (13)
Low C3	8 (14.5)
Low C4	6 (10.9)
Low C3 and C4	4 (7.2)

Note: Values are presented as a number (%). Abbreviations: aCL, anticardiolipin antibody; aPL, antiphospholipid antibody; aβ2GPI, anti-β2-glycoprotein I; CVD, cardiovascular disease; LA, lupus anticoagulant; PEC, preeclampsia; PI, placental insufficiency; VTE, venous thromboembolism.

**Table 5:** Comparison of patients with and without recurrent thrombosis in APS.

	Recurrent Thrombosis (n = 17)	No Recurrent Thrombosis (n = 38)	*p*-Value
Age (years)	41.82 ± 11.89	38.66 ± 12.39	0.380
Disease duration (months)	60 (12–120)	12 (12–30)	0.028
Smoking, n (%)	4 (23.5)	11 (28.9)	0.754
Comorbidities, n (%)			
Arterial hypertension	7 (41.2)	11 (28.9)	0.340
Diabetes mellitus	2 (11.8)	3 (7.9)	1.000
ASCVD	3 (17.6)	2 (5.3)	0.314
Hyperlipidemia	1 (5.9)	5 (13.2)	0.650
aGAPSS	6.53 ± 2.62	8.00 ± 3.57	0.095
Clinical domain, n (%)			
Venous thrombosis	13 (76.5)	20 (52.6)	0.095
Arterial thrombosis	7 (41.2)	17 (44.7)	0.806
Livedoid vasculopathy	1 (5.9)	6 (15.8)	0.416
Obstetric	4 (23.5)	10 (26.3)	1.000
Thrombocytopenia	5 (29.4)	14 (36.8)	0.592
Laboratory results, n (%)			
Triple aPL positivity	0 (0)	7 (18.4)	0.419
Positive LA (One time)	1 (6.3)	3 (7.9)	1.000
Positive LA (Persistent)	13 (81.3)	30 (78.9)	1.000
Moderate or high positive (IgM) (aCL and/or aβ2GPI)	1 (5.9)	5 (13.5)	0.652
Moderate positive (IgG) (aCL and/or aβ2GPI)	3 (17.6)	12 (32.4)	0.338
High positive (IgG) (aCL or aβ2GPI)	3 (17.6)	1 (2.7)	0.087
High positive (IgG) (aCL and aβ2GPI)	1 (5.9)	6 (16.2)	0.412
Low C3	4 (23.5)	4 (10.5)	0.210
Low C4	2 (11.8)	4 (10.5)	1.000
Low C3 and C4	2 (11.8)	2 (5.3)	0.588
25(OH)D level, (ng/mL)	17.58 ± 13.50	14.05 ± 8.74	0.250

Note: Data are presented as mean ± SD, median (min–max), or number (%), as appropriate. Abbreviations: aCL, anticardiolipin antibody; aPL, antiphospholipid antibody; ASCVD, atherosclerotic cardiovascular disease; aβ2GPI, anti-β2-glycoprotein I; LA, lupus anticoagulant; Bold *p*-values indicate statistical significance (*p* < 0.05).

**Table 6:** Multivariable logistic regression analysis for recurrent thrombosis in APS patients.

Variable	OR	95% CI	*p*-Value
Age (years)	1.01	0.95–1.06	0.676
Disease duration (months)	1.00	0.99–1.01	0.141
aGAPSS	0.90	0.73–1.10	0.320

Abbreviations: OR: odds ratio; CI: confidence interval; aGAPSS: adjusted Global Antiphospholipid Syndrome Score. Variables included in the multivariable model were selected based on clinical relevance, literature support, and univariate analysis results.

## Data Availability

The data underlying this article will be shared on reasonable request by the corresponding author.
